# Vocal plasticity in harbour seal pups

**DOI:** 10.1098/rstb.2020.0456

**Published:** 2021-12-20

**Authors:** Laura Torres Borda, Yannick Jadoul, Heikki Rasilo, Anna Salazar Casals, Andrea Ravignani

**Affiliations:** ^1^ Comparative Bioacoustics Group, Max Planck Institute for Psycholinguistics, Wundtlaan 1, 6525 XD Nijmegen, The Netherlands; ^2^ Research Department, Sealcentre Pieterburen, Hoofdstraat 94-A, 9968 AG Pieterburen, The Netherlands; ^3^ Artificial Intelligence Lab, Vrije Universiteit Brussel, 1050 Elsene/Ixelles, Belgium

**Keywords:** vocal modulation, vocal production learning, volitional control, pinniped, acoustic communication

## Abstract

Vocal plasticity can occur in response to environmental and biological factors, including conspecifics' vocalizations and noise. Pinnipeds are one of the few mammalian groups capable of vocal learning, and are therefore relevant to understanding the evolution of vocal plasticity in humans and other animals. Here, we investigate the vocal plasticity of harbour seals (*Phoca vitulina*), a species with vocal learning abilities observed in adulthood but not puppyhood. To evaluate early mammalian vocal development, we tested 1–3 weeks-old seal pups. We tailored noise playbacks to this species and age to induce seal pups to shift their fundamental frequency (*f*_0_), rather than adapt call amplitude or temporal characteristics. We exposed individual pups to low- and high-intensity bandpass-filtered noise, which spanned—and masked—their typical range of *f*_0_; simultaneously, we recorded pups' spontaneous calls. Unlike most mammals, pups modified their vocalizations by lowering their *f*_0_ in response to increased noise. This modulation was precise and adapted to the particular experimental manipulation of the noise condition. In addition, higher levels of noise induced less dispersion around the mean *f*_0_, suggesting that pups may have actively focused their phonatory efforts to target lower frequencies. Noise did not seem to affect call amplitude. However, one seal showed two characteristics of the Lombard effect known for human speech in noise: significant increase in call amplitude and flattening of spectral tilt. Our relatively low noise levels may have favoured *f*_0_ modulation while inhibiting amplitude adjustments. This lowering of *f*_0_ is unusual, as most animals commonly display no such *f*_0_ shift. Our data represent a relatively rare case in mammalian neonates, and have implications for the evolution of vocal plasticity and vocal learning across species, including humans.

This article is part of the theme issue ‘Voice modulation: from origin and mechanism to social impact (Part I)’.

## 1. Introduction

### (a) Animal communication and plasticity

In many species, accurate communication is crucial: it can increase potential mating opportunities, the probability of escaping from a predator, and the speed of social learning [1]. Biotic and abiotic factors can both impact communication. Acoustic communication is particularly developed in marine mammals because of the selection pressures of the marine environment [2,3]: underwater sounds propagate over long distances, whereas water clarity or light level can limit transmission of chemical or visual cues [4]. However, noise can lead to signal degradation, and when the frequency range of a signal overlaps with the frequencies of noise the signal gets masked [5].

Being vocally plastic allows individuals to adjust their vocal signals in response to changes in their environment [5]. Plasticity, if present in a species, occurs in various contexts. In particular, interferences in signal detection can lead to important adaptations underlying the evolution of animal communication systems: vocal plasticity enables some animals, including humans, to reach their communicative goal, potentially via different mechanisms.

### (b) The Lombard effect

Many animal species increase the amplitude levels of their vocalizations in the presence of masking noise to ‘sound louder' [6–9], especially when the noise overlaps with the spectral composition of the species-typical vocalization [10,11]. This vocal adjustment is often referred to as the Lombard effect [12], and serves to increase the signal-to-noise ratio (SNR) in vocal communication under noise. This well-studied type of signal modification requires little plasticity and is common across species.

In human communication, the Lombard effect is more prominent when speaking occurs with communicative intent and with a speaking partner than when speaking aloud alone [13]. The type of voice modification and its strength vary between individuals [14]. The Lombard effect, quantified as vocal sound pressure level (SPL), starts to take place at 43.3 dB(A) of pink background noise, after which the SPL of the speaker increases by 0.65 dB(A) per 1 dB(A) of added noise level [15]. Within the Lombard effect, a flattening of the spectral tilt produces a significant increase of speech intelligibility under noise. Conversely, an increase in fundamental frequency (*f*_0_) does not lead to a significant increase in intelligibility [16], and thus might be a side-effect of increased subglottal pressure to achieve higher call amplitudes [17,18].

Amplitude adjustment is only one, extremely common, adaptive strategy previously observed in several bird species [19], marine mammals [20–22], bats [23] and primates [11]. When flexibly adapting their vocal output, some species exhibit rare spectral changes [24], while others show different vocal behaviours, such as temporal shifts (e.g. changes in call rate or duration) [25–27].

### (c) Spectral adjustments in animal communication

Parallel strands of research investigate vocal production learning, which is the ability to modify species-specific vocalizations or create novel ones, often through imitation [28]. Vocal learning can also arise from plastic adaptations to environmental factors, but complex forms of vocal plasticity involve modulation of *f*_0_ or formants. These are often, but not always, supported by control of vocal articulators and oral cavities [29].

Anatomical adaptations and emotional contexts can affect a species' *f*_0_ with no need for plasticity or control. By contrast, mammals rarely display volitional modulation of vocal parameters, such as *f*_0_. Studies have demonstrated the capacity of *f*_0_ modulation [28,30] through vocal imitation in some species (elephants: [31,32]; bats: [33]). However, evidence of *f*_0_ shifts while facing environmental noise is limited. Adjusting *f*_0_ under noisy conditions might illustrate the animal's motivation to adapt *f*_0_ also to achieve accurate communication, rather than only imitating experimental stimuli. Birds have been shown to increase their *f*_0_ in urban environments due to low frequency traffic noise [34]. However, *f*_0_ shifts due to noise are considered rare in animals and, when they occur, may be driven by the Lombard effect as a physiological by-product of higher vocal amplitude [35].

### (d) Our approach

In the current study, we investigated vocal plasticity of harbour seal pups (*Phoca vitulina*). This is especially important for one reason: some adult pinnipeds are capable of vocal production learning [36,37]. Among mammals, pinnipeds are an excellent model for vocal learning: they are phylogenetically closer to humans than other classical model species (e.g. songbirds) and exhibit a variety of spontaneously produced vocalizations [36,38,39]. However, harbour seal adults are relatively silent, with males being mostly vocal during the breeding season [40].

Here, we attempted to combine the best of two contrasting empirical approaches. Previous studies in a laboratory setting have shown the advantages of an experimentally controlled environment, but also the challenges of obtaining spontaneous vocalizations [11,41,42]. Operant conditioning techniques to elicit vocalizations have proved effective, yet it can be difficult to disentangle natural predispositions towards a task from learning attitudes [11,24,27,43]. At the other extreme, fieldwork with wild animals favours naturalness and spontaneity, sometimes at the expense of experimental control. Here, we tested wild animals soon after they reached captivity, while also capitalizing on their natural proclivity to spontaneously produce vocalizations.

Within marine mammals, previous studies targeting amplitude shifts in the presence of background noise have mainly focused on cetaceans, among them bottlenose dolphins and humpback whales [20–22,25,44]. Within phocid pinnipeds, bearded seals have been shown to increase the amplitude of their underwater calls in higher ambient noise conditions [45]. To our knowledge, the only studies in harbour seals targeted adult male underwater vocalizations, finding little to no adjustment to noise [46]. By contrast, we decided to investigate the plasticity of *f*_0_ in seal pups. In natural conditions, the *f*_0_ range of harbour seal pups varies between 270 Hz and 620 Hz, and a gradual downward change is observed in males throughout their vocal development [47].

### (e) Hypotheses and predictions

We aimed at triggering shifts in *f*_0_ (and potentially other vocal parameters) in a controlled experimental setting. Our goal was to induce volitional spectral shifts, produced as a strategy to avoid acoustic masking in a noisy environment, thus illustrating unusual vocal plasticity in this promising taxon. While *f*_0_ was our main experimental target, we considered the possibility that seals may also adjust other parameters in response to masking.

We first hypothesized that seal pups would shift their *f*_0_ upwards or downwards to escape the bandpass-filtered noise that we purposely tuned to overlap with their *f*_0_ range. Alternatively, a lack of *f*_0_ shift could support hypotheses of less reliance on *f*_0_ adaptations in social communication, or lack of the vocal plasticity necessary to conduct such modulations.

Second, if seals behaved similarly to other species, we would expect to observe temporal shifts [19,26,48,49]. According to this hypothesis, during noisy periods pups would vocalize more and longer compared to the absence of playback [26].

Finally, if pups showed a typical Lombard effect, we would expect them to increase their vocalizations' amplitude during playbacks of lower-intensity noise versus no playbacks, and even more so during higher-intensity noise. Conversely, no amplitude shifts would suggest that higher noise levels may be needed or that seals adopt a different strategy in response to masking.

Overall, there could be a trade-off between vocal adjustments, leading to various changes. While a lack of both frequency and amplitude shifts would confirm the results obtained in adult seals [46], an amplitude-only shift would point towards a general Lombard mechanism. A simultaneous upwards shift in both could suggest the *f*_0_ shift to be a mechanical by-product of the amplitude shift. Finally, a frequency-only shift may point towards vocal plasticity possibly due to good neural control of the larynx.

## 2. Material and methods

### (a) Subjects and study site

The study was conducted at the Sealcentre Pieterburen, a seal rehabilitation centre specialized in phocids [27,50]. The Sealcentre rescues a yearly average of 400 seals (family Phocidae), which are later released back into the wild. Tested seal pups were housed in quarantine units, where all recordings were performed with no water in the pool, hence avoiding water noises. Data collection started immediately after arrival of the individuals and once the Sealcentre's veterinarians confirmed that the animals were not suffering from any extenuating disease (electronic supplementary material, table S1). Following the centre's policies, access to the animals was only possible during set time windows (four daily feeding times), which were decided independently from the experiment.

We tested 8 wild-born, Eastern-Atlantic harbour seal pups. This species is monotocous, ensuring the animals could not be siblings. The veterinarians estimated the pups' age during the first veterinary examination following the Sealcentre's protocols [51]: the 8 tested seal pups (three females) were all aged between 7 and 10 days on their first day of testing. They were housed in pairs, each pair in an independent quarantine area (electronic supplementary material, figure S1). Housing conditions of all four rooms were identical.

Data collection was non-invasive, was approved by the centre's veterinarians and adhered to the guidelines of the Association for the Study of Animal Behaviour [52]. We observed that the noise playback did not increase the pups' behavioural indicators of stress by live video monitoring the first playbacks under the supervision of the research and veterinary team.

### (b) Stimuli

Playbacks were based on audio recordings of ambient noise, mostly wind sounds, from a sandbank in the Wadden Sea (see electronic supplementary material). Sounds were bandpass-filtered in Praat (v. 6.0.52; [53]), resulting in a noise band between 250 and 500 Hz. This frequency range was chosen to overlap with the *f*_0_ range of seal pups' mother attraction calls [47,54,55].

An experimental session consisted of playback of a 45 min audio file (WAVE format) composed of three sequences of 5-min *high noise* (65 dB SPL), three sequences of 5-min *low noise* (45 dB SPL) and three sequences of 5 min with *no playback* (resulting in approx. 25 dB SPL of background noise). Prior to experimental trials, noise playbacks were measured with an SPL meter positioned in the centre of the dry pool at a seal pup's ear height (approx. 30 cm). The order of the nine sequences within playbacks was randomized for each seal pair and experimental session, while making sure to avoid two identical noise intensity conditions in a row.

### (c) Apparatus and experimental procedure

Sounds were broadcasted via a Yamaha HS5 Speaker. Recordings were performed with a unidirectional microphone Sennheiser ME-66 on a tripod. During playback, this microphone, connected to a Zoom F8 recorder, recorded the pups' vocal responses. The apparatus was located approximately 2 m from the pup at one corner of the pool (electronic supplementary material, figure S1).

We tested four pups per day (one session a day in two units). Once we obtained seven valid sessions (a valid session being one containing at least two vocalizations), we proceeded to testing four additional pups. The first unit was tested at 14:15 and the second one at 18:15. These times were chosen, in agreement with the Sealcentre's veterinarians, to increase the likelihood of successfully recording spontaneous vocalizations because pups are usually more vocal before feeding. The apparatus was temporarily installed in each unit 3 h before each session (i.e. 11:00 and 15:00) and directly removed after. By the end of the study, all animals had been recorded between 10 and 14 times to reach seven valid sessions.

### (d) Sound recordings, annotations and *f*_0_ extraction

A Zoom Q8 handy video recorder filmed every trial. Because two individuals were housed in the same unit, one of them was marked with an animal-coloured marker. Audio and video recordings were synchronized to assign each vocalization to the pup that produced it using BORIS [56].

The onset and offset of each vocalization in the recorded files (WAVE format) were manually annotated in Praat v. 6.0.52 [53]. Acoustic analyses were carried out in Python and Matlab. Specifically, after annotation, Parselmouth (a Python library for Praat; v. 0.3.3, Praat v. 6.0.37; [57]) was used to extract duration and *f*_0_ of the annotated calls (autocorrelation method for pitch tracking, with non-default parameters: time step 0.01 s, pitch floor 200 Hz and pitch ceiling 800 Hz). All calls were included in the analyses of the number of calls and their duration. However, only calls that (1) were not clipped, (2) did not overlap with other individuals, (3) did not contain background noise other than the playback and (4) could be properly tracked by Praat, were included in the analyses of calls' amplitude and *f*_0_.

Praat's ability to track the pitch in all noise conditions was checked manually on a large random sample of calls. This was first done by zooming in on the sound wave, selecting a single period, and calculating the frequency as the inverse of its wavelength. Spectrograms were then visually verified, checking whether estimates by Praat matched the *f*_0_ and harmonics in the spectrogram. By doing so, we did not find any bias due to the pitch-tracking algorithm's performance in our recordings: even in cases where the high-intensity noise condition obscured the *f*_0_ in the 250–500 Hz frequency band, the harmonics provided enough autocorrelation information for Praat's algorithm to estimate *f*_0_.

### (e) Amplitude and spectral tilt

We obtained average spectra and intensity values for each call to test if seals adjusted their vocalizations' amplitude or spectral tilt depending on the noise condition. To account for the differential contribution of noise in each condition, separate recordings were made of noise only (seals not present) with otherwise equal recording set-up. The intensity and spectral characteristics of the background noise were seen to vary slightly over time, and noise-only recordings enabled more accurate estimation of the noise characteristics during each vocalization. Due to the very reverberant recording conditions and additional noise sources (e.g. bird and airplane sounds), perfect cancellation of the playback noise from each recording was not possible. For comparisons between conditions, we tried to reduce the effect of the noise based on spectral subtraction by subtracting the averaged power spectrum of the estimated background noise from that of the vocalization [58]. Background noise increased the mean and the variance of the spectral content of the underlying calls. Spectral subtraction can recover the mean spectral content but the variance will remain distorted by the noise variance [58].

Each recording session had slightly varying preamplifier gain in the recording phase due to manual adjustment. This gain variation was compensated by (1) calculating the root-mean-square (RMS) power for each noise condition from each recording from the moments when the seals were not vocalizing and (2) determining a gain value per recording session. This compensation brought the average power of the low and high noise conditions to the same level as the corresponding average value in the noise-only recordings.

To perform call amplitude analysis, we calculated the RMS power of each call (RMS power is proportional to the RMS sound intensity, and for simplicity, this measure will be called *intensity* from here on). Similarly, we calculated the intensity of the noise-only recording from the corresponding location and subtracted it from the intensity of the call. If the SNR of a call was too low, this subtraction could lead to a negative intensity value for the call. However, as the mean over all calls after the subtraction should represent the mean of the original calls, comparisons in the linear (non-decibel) domain were possible.

The Lombard effect on human speakers shows as an energy boost on high frequencies. This can be characterized by, for example, a flattening of the spectral tilt. In [16,59], speech in noisy conditions showed as a spectral energy boost between 0.5–1 kHz and 5 kHz when compared with the silent condition. There is high variance between different ways of measuring spectral tilt [60]; thus, here, spectral tilt was estimated using two separate methods.

First, after spectral subtraction, the spectral slope was calculated by fitting a regression line in the log-energies on ⅓ octave frequencies, as done in [16]. In this work, 1 kHz was used as a reference frequency for the ⅓ octave filters, and the line was fitted only on frequencies above 400 Hz. We adopted this cut-off because the *f*_0_ of the vocalizations lay around this frequency, and the background noise mostly corrupted estimates of the spectral energy below 500 Hz. The regression line was fitted only on the octave energies whose values remained positive after spectral subtraction. For 11 calls, octave slope could not be estimated due to lack of positive energy value on two or more bins after spectral subtraction. These occurred only in the high-noise condition, and were discarded from the spectral slope analysis.

Second, again after spectral subtraction, a ratio of the spectral energy between 0.4 and 1 kHz to that of 1–4 kHz was calculated (R14). This method was adjusted from [61]: instead of considering all energy below 1 kHz as in their work, we removed energies below 400 Hz from the analysis as justified above. For spectral analysis of each call and corresponding noise estimated from the noise-only recording, the average power spectra were calculated using fast Fourier transform (FFT) with window size of 512 samples, overlap of 256 samples and Hamming windowing.

### (f) Statistical analysis

The effect of the noise intensity on the number of vocalizations, call duration and *f*_0_ was analysed by fitting a linear mixed-effects model. These extracted acoustic parameters were included as dependent variables, predicted by the background noise condition as an independent variable (factor with three levels: no playback, low noise and high noise). The session number (seven sessions per pair) and the specific seal identity were modelled as random intercept effects. We also included a variable named ‘trial number' as a fixed effect predictor to control for the existence of a learning or habituation effect within sessions. This variable allowed us to test whether changes in vocal behaviour were affected by the time course of the session.

Statistical analyses were performed in R, v. 3.5.2 [62]. Comparisons were done with linear mixed-effects models using the package nlme [63]. *p*-values were calculated via Monte Carlo sampling with 1000 permutations using the PermTest function of the R package pgirmess [64]. Permutation tests for linear models were chosen because they suited our limited sample size and relaxed the assumption of normality of residuals [65]. Moreover, a Bonferroni adjustment for multiple comparisons was applied to all pairwise comparisons. Significance was set at *p* < 0.05/3 (≈ 0.0167). When summary statistics are reported in the results, these are condition means or medians, rather than model estimates.

To analyse the effect of the intensity of playback noise on the intensity of the seals' vocalizations and the two spectral tilt measures described above, we used the non-parametric Mann–Whitney U test. These three variables were analysed differently from the previous ones, since they were strongly non-normally distributed and as such could not be fitted by linear models. Moreover, this statistical analysis allowed us to more straightforwardly investigate each seal separately, as the average spectra per seal (see electronic supplementary material) indicated a difference between individuals. To account for the individual effect of seals, tests were done per seal. To correct for multiple comparisons, we applied a Bonferroni correction of 24 (8 seals, 3 pairwise comparisons between the three noise conditions), resulting in a required significance level of *p* < 0.05/24 (≈0.00208).

## 3. Results

We recorded a total of *N* = 3534 calls. We tested 8 pups and obtained seven valid sessions per pair (mean range from first to last valid recording day: 10.75 days, min: 9 days, max: 13 days). Statistical analyses conducted on vocalizations' amplitude and *f*_0_ were performed over 2576 ‘clean' calls (see the four criteria in §2d). Statistical analyses on vocalizations' rate and duration were performed over the totality of recorded calls.

Briefly summarized, we found a significant effect of the noise condition on *f*_0_, with seals producing calls with lower *f*_0_ the noisier the condition. A few seals also showed varying degrees of modulation of call amplitude and spectral tilt depending on the noise condition, possibly due to the Lombard effect.

### (a) Number of calls and duration

**Figure 1.**
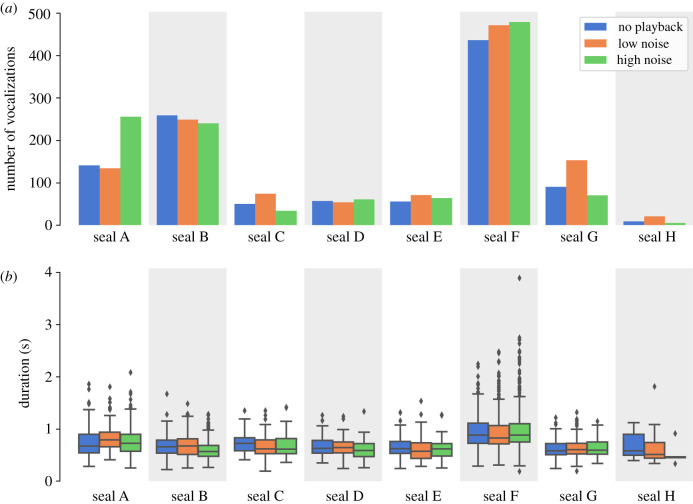
Plots of the number of calls per seal (*a*) and the vocalizations' duration distributions (*b*) show there is some inter-seal variation. However, we found no consistent, significant effect among the three conditions.

Noise conditions did not significantly affect the number of vocalizations (pseudo*R*^2^ = 0.027; *p* = 0.341; *N* = 245). Thus, pups did not significantly increase or decrease their number of vocalizations depending on the noise intensity (figure 1*a*). Seals produced 1209 calls during high noise, 1227 calls during low noise and 1097 calls during no playback. The numbers of vocalizations were also comparable throughout the conditions.

Noise conditions also did not affect calls' duration (figure 1*b*). Vocalizations were neither significantly longer nor shorter as noise level increased (pseudo*R*^2^ = 0.014; *p* = 0.707; *N* = 3534). Pups' calls lasted 0.785 s on average (median: 0.729 s; min: 0.182 s; max: 3.892 s).

### (b) Fundamental frequency (*f*_0_)

**Figure 2.**
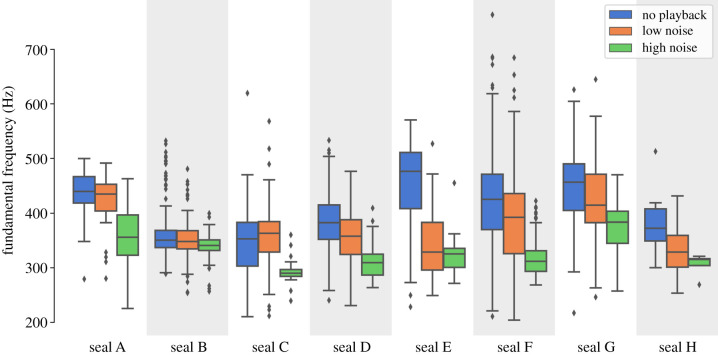
The three different conditions of noise intensity had a significant effect on the *f*_0_ of the seals' vocalizations, with increased noise leading to a lower *f*_0_.

We tested the effect of the noise condition on *f*_0_ (figure 2). A main significant effect was found (pseudo*R*^2^ = 0.202; *p* < 0.001; *N* = 2576). Pairwise comparisons showed significant differences between our three levels. In high noise, *f*_0_ was significantly lower than in low noise (pseudo*R*^2^ = 0.166; *p* < 0.001; *N* = 1751) and no playback (pseudo*R*^2^ = 0.287; *p* < 0.001; *N* = 1636). *f*_0_ was also significantly lower in low noise than in no playback (pseudo*R*^2^ = 0.038; *p* < 0.001; *N* = 1765). The *f*_0_ median was equal to 324 Hz in the high noise condition, 374 Hz in the low noise condition and 403 Hz in the no playback condition.

Because of our playback duration, we could have observed differences in vocal behaviour among trials, i.e. between the beginning and the end of each session due to habituation, frustration or tiredness. We did not find any significant effect of trials on *f*_0_ (*p* = 0.184; *N* = 2576).

### (c) Amplitude and spectral tilt

**Figure 3.**
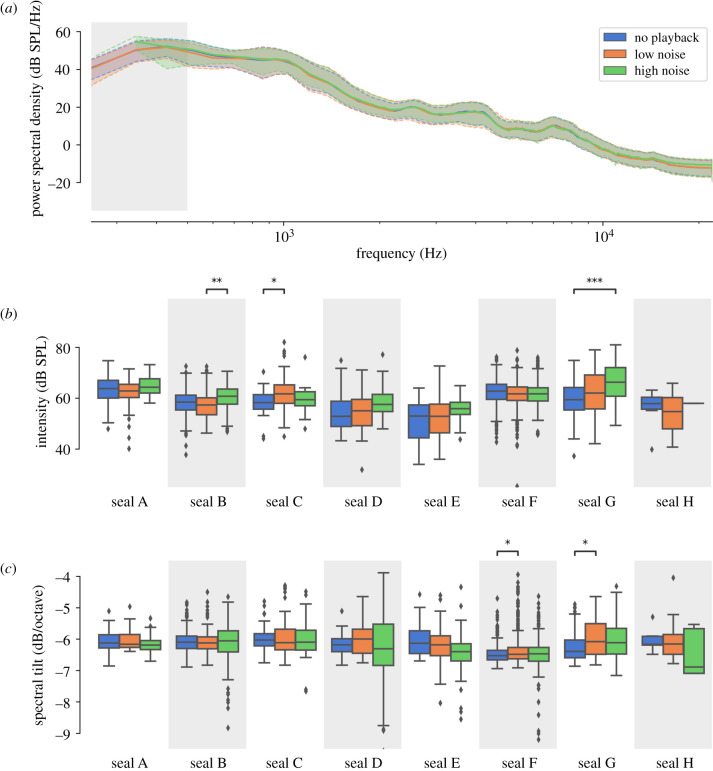
The median spectrum over all seal vocalizations grouped per noise intensity level and its first and third quartile (*a*) illustrate the lack of general effect of the noise on the seals' vocalizations—see also electronic supplementary material, figure S2 for individual spectra. There was no overall effect of the noise levels on the intensity of vocalizations after compensating for the noise intensity (*b*) either, but seal B, seal C and in particular seal G showed a significant increase in their vocalizations' intensity between at least two conditions. Similarly, the fitted slopes of the spectral tilts (*c*) of seal F and seal G's average vocalization spectra show a flatter spectral tilt in noisier conditions and provide suggestive indication of the Lombard effect potentially occurring in these individuals. Significant differences between conditions in *b* and *c* are marked with asterisks (*p < 0.05; **p < 0.01; ***p < 0.001; *p*-values are Bonferroni-corrected by factor 24).

Initial Mann–Whitney U tests on the whole dataset showed no significant effects on call intensity (see also figure 3*a*; electronic supplementary material, table S2). After Bonferroni-correction for two measures, both spectral tilt measures showed an effect only between no playback and low noise condition (no playback versus low noise: R14: *p* = 0.0041, slope: *p* < 0.001; no playback versus high noise: R14: *p* = 0.025, slope: *p* = 0.47). After analysing calling patterns of individual seals, one seal appeared to contribute most to the seen global effect^1^ (figure 3*b*). Follow-up analyses were performed for each seal separately.

Seal G showed increased call intensity with increasing noise level. The effect between no playback and high noise condition was significant (*p* < 0.001). The spectral slope flattened by 0.31 dB/octave from no playback to low noise (*p* < 0.001; figure 3*c*). Similarly, the spectral ratio R14 (electronic supplementary material, figure S3) decreased from no playback to low noise (*p* < 0.001) and no playback to high noise (*p* = 0.0011). Seal C showed a significant effect in intensity between no playback and low condition (*p* = 0.0012), but no other significant effects following the Lombard hypothesis. Seal B showed a significant effect in intensity (*p <* 0.001) and R14 (*p* = 0.0019) only between the low noise and high noise conditions.

### (d) Coefficient of variation

Statistical analyses on the level of dispersion around the mean were conducted for vocalizations' duration and *f*_0_ (figure 2). We did not calculate dispersion for the number of calls, which is already a summary statistic, and for the intensity measurements, which followed a highly skewed, non-normal distribution (cfr. §§2e,f). We calculated the coefficient of variation of vocalizations grouped by session, seal identity and condition. No significant differences between conditions were found on the coefficient of variation of calls' duration (pseudo*R^2^* = 0.002; *p* = 0.886; *N* = 118). However, we found a significant effect of noise conditions on the coefficient of variation of *f*_0_ (pseudo*R^2^* = 0.195; *p* < 0.001; *N* = 109). Pairwise comparisons showed that the coefficient of variation of calls was significantly lower in the high noise condition compared to both low noise (pseudo*R*^2^ = 0.256; *p* < 0.001; *N* = 69) and no playback conditions (pseudo*R*^2^ = 0.233; *p* < 0.001; *N* = 71). No significant difference was found between the low noise and no playback conditions (pseudo*R*^2^ = 0.004; *p* = 0.510; *N* = 78).

## 4. Discussion

### (a) Overview of findings

Our data showed a clear downward *f*_0_ shift in pups' calls in response to noise masking, which was the original purpose of this experiment. The number of calls and their duration were neither influenced by the presence of ambient noise nor by its intensity levels. In addition, three out of eight pups showed limited modulation of their call amplitude depending on the noise condition, perhaps indicating compensation for acoustic masking.

Overall, we found no strong modulation of spectral tilt or call amplitude as a response to increased noise levels. Both of these quantities are usually measured when testing for the Lombard effect in human speech and animal vocalizations. These amplitude findings are in line with previous results where adult harbour seals did not significantly increase their call amplitude in response to noise [46].

Recent evidence from animal studies shows that the SNR between animal vocalizations and background noise is a better predictor for the Lombard effect than the ambient noise level alone [66]: the lower the SNR, the more likely the Lombard effect may be to appear. In our study, we estimated the SNR experienced by the seal pups in the high noise condition to be roughly +10 dB on average.^2^ For comparison, it has been shown that SNRs of −5 to −20 dB induced the Lombard effect in frogs, whereas an SNR of +20 dB did not [66]. The underwater vocalizations of harbour seals in [46] had a very high SNR (50–70 dB), perhaps also contributing to the lack of observed Lombard effect. However, bearded seals did increase their call amplitudes under higher ambient noise with similarly high SNR [45]. Garnier & Henrich [59] reported how the Lombard effect on human speakers helped maintain a +12.5 dB SNR under noise conditions on average, where the SNR would otherwise be negative without any vocal intensity modification. Drawing on this evidence, the observed lack of general amplitude shift in our experiment may be due to the high SNR, caused by relatively low playback noise levels. Another explanation could be that seal pups always vocalize close to their physical limits, and thus cannot adjust to different noise conditions (see also [45,46,67]).

### (b) Amplitude modulation in one pup

One seal pup showed a peculiar vocal behaviour. Vocalizations of pup G showed (1) flattening of the spectral tilt when background noise was present and (2) increasing intensity as noise increased. Energy of the vocalizations on the 1–4 kHz spectral band increased more than that of below 1 kHz in response to noise, similarly to the Lombard effect in human speakers. The flattening in the spectral tilt observed for pup G under noise was approximately 0.31 dB/octave (no additional flattening from low to high noise condition). A similar change (flattening of 0.27 dB/octave) in spectral tilt occurs in human speakers speaking in quiet versus 82 dB SPL background noise [16]. Our average +10 dB SNR in the high noise condition may be close to the threshold where the Lombard effect begins to take place. In addition to the strong evidence for seal pup G, this may also explain the sporadic effects of spectral tilt and amplitude modulation for pups B, C and F.

The noise threshold inducing the Lombard effect is variable among humans [14]. If this generalizes to other species, pup G may have been more responsive to noise compared to the other individuals. Furthermore, its vocal behaviour could illustrate a higher motivation and arousal induced by the noise context. More speculatively, pup G's behaviour may have arisen from stronger communicative intent compared to the other pups [13]. Based on these results, we cannot exclude that seal pups can increase the amplitude of their voices in response to noise.

### (c) Fundamental frequency shift

Our experimental playback successfully incited the seals to modify their vocal production, especially our main parameter of interest: *f*_0_. This behaviour may be an adaptation to avoid spectral masking of one's *f*_0_. Pups' vocal modification was precise in time and adapted to the particular noise broadcasted during this experiment. Our results show that seal pups modified their vocalizations in a unique way: a downwards *f*_0_ shift was observed in response to increased ambient noise. The lowering of *f*_0_ is atypical when compared to other species that have shown either no shift or an increase in their *f*_0_ [5,35,68] (see [68] for a case of *f*_0_ downward shift, notably in another mammalian vocal learner). Analyses on the dispersion of *f*_0_ around the mean across vocalizations revealed that dispersion was lower in the high noise condition than the low noise condition and no playback condition. This suggests that, in addition to shifting down their *f*_0_, seal pups may have *focussed* their vocal production towards these lower frequencies. This downward shift of *f*_0_ could have at least two functional explanations. First, it may be an adaptation to the actual environmental noise that pups encounter: as lower frequencies propagate better in wind, shifting *f*_0_ downwards may increase the travel distance of calls [69]. Second, lowering of the *f*_0_ may be a way for seal pups to better communicate their identity. As a low *f*_0_ induces closely spaced harmonics, hence more frequencies per frequency band, the upper vocal tract acting as a filter has a ‘denser' source to create formants on. Close spacing of harmonics contributes to enhanced formant information [70,71], which may be a key parameter for individuality encoding [72,73].

The shift in *f*_0_ cannot be explained by automatic adaptations (as opposed to some vocal control). Indeed, arousal can lead to tension of the vocal folds, inducing an increase in vibration frequency and producing, in turn, an increase in *f*_0_ [74]. The downwards *f*_0_ shift we find therefore contrasts with predictions of arousal-driven *f*_0_ shifts. Our evidence in harbour seal pups may be interpreted as a behavioural proxy for advanced laryngeal control in this species.

### (d) Vocal plasticity and neuro-anatomical mechanisms

Vocal production involving volitional modulations of acoustic parameters may highlight a rare ability of vocal plasticity in harbour seal pups. It has been previously shown that elaborate control over the vocal apparatus provides a biophysical mechanism for vocal learning. Thus, laryngeal plasticity and vocal flexibility may provide indirect evidence for vocal learning in harbour seals' puppyhood [28,38,75].

*f*_0_ is a major feature shaping human singing and speech production. Frequency modulations may be physiologically more demanding to perform than temporal or amplitude modulations; in fact, several anatomical and dynamic features affect *f*_0_, such as length, tension and stiffness of the vocal folds [38,76]. Therefore, controlling *f*_0_ requires neuromuscular control over several anatomical structures whereas duration and amplitude of a sound are mostly controlled by modifications of exhalation.

In humans, vocal learning requires control—mediated by the laryngeal motor cortex—over multiple phonatory structures linked to both the source and the filter [77,78]. Neurobiological studies, based on electrical stimulation and localized destructions, showed that the laryngeal motor cortex has a key role in volitional control of vocal fold movements [79,80]. Direct cortico-bulbar connections have been suggested to be the main anatomical explanation for humans' capacities of fine laryngeal control and vocal plasticity [81–83]. By contrast, non-human apes have only limited direct connectivity [83], and some mammals incapable of vocal learning possess only *indirect* cortico-bulbar connections for laryngeal-motor neurons. This neuro-anatomical difference could explain the limited vocal plasticity of non-vocal learners [84,85]. Supporting the direct connections hypothesis, songbirds with vocal learning abilities might have direct connections analogous to humans [86]. To date, there is no evidence for direct cortico-bulbar projections for these specific neurons in any mammalian species except for humans. Our behavioural results lead to the prediction that harbour seals would be prime candidates among mammals to show direct anatomical connectivity between the laryngeal motor cortex and laryngeal motoneurons, as seen in humans [81,87].

### (e) Future work and conclusions

Additional research on the level of control seals might exert over different parts of their phonatory apparatus can shed light on fine-grained mechanisms for vocal learning. Considering our relatively straightforward setup, we suggest that *f*_0_ modulation in response to noise could be a powerful cross-species test (see e.g. similar work in bats [5,68]) for the presence of vocal learning abilities and actual degree of vocal plasticity, testing their association.

Further studies could investigate whether the modulation of *f*_0_ in the presence of spectral masking is biologically relevant and actually perceived by conspecific harbour seals. In addition, future replications of the current experiment could test the effect of louder playbacks on the amplitude shift [15] and apply various masking frequency bands to test whether different frequencies could induce an upward versus downward shift in *f*_0_. As a complement to behavioural experiments, anatomical work could investigate the elastic properties of seal larynges to establish upper and lower anatomical boundaries for *f*_0_ production [50,88]. Finally, neurobiological work should track purported direct cortico-laryngeal connections in seal pups, and compare them against closely related Caniformia not capable of *f*_0_ plasticity [78,87].

To conclude, our data show plastic vocal behaviour in a neonate mammal, similar to that of humans and very few other adult mammals [5,68]. As we learn more about vocal plasticity across species, we will be able to construct acoustic phylogenies of this trait in mammals. This will shed light not only on how environment and ancestry interact to deliver adaptable communication, but indirectly provide information on the evolution of speech and song in our own species.

## Footnotes

1 Entirely analogously to the previous statistical analyses, we also fit a mixed-effect linear model with the seals as random effects. The noise condition was only found to have a significant effect on the spectral tilt (*p* = 0.038), and *post hoc* tests showed a significant difference between the no playback and low noise conditions (*p* = 0.002). As such, the results are comparable to those of the global Mann–Whitney U tests. However, inspection of the individual seals’ spectra (see electronic supplementary material) warrants a statistical analysis per individual seal.

2 Assuming the pup was in the middle of the pool, the noise level in the high noise condition at the position of the pup was 65 dB, whereas the mean intensity of calls at the position of the microphone (at 2 m distance from the pup) was 59.7 dB in the no playback condition, and 60.3 dB in the high noise condition after spectral subtraction. Using the inverse square law, we estimate the average call amplitude at the position of the pup to be roughly 75 dB.

## Data accessibility

Data have been uploaded as electronic supplementary material [89].
